# Continuous exposure of pancreatic cancer cells to dietary bioactive agents does not induce drug resistance unlike chemotherapy

**DOI:** 10.1038/cddis.2016.157

**Published:** 2016-06-02

**Authors:** P Fan, Y Zhang, L Liu, Z Zhao, Y Yin, X Xiao, N Bauer, J Gladkich, J Mattern, C Gao, P Schemmer, W Gross, I Herr

**Affiliations:** 1 Molecular OncoSurgery, University of Heidelberg, Heidelberg, Germany; 2 Section Surgical Research, University of Heidelberg, Heidelberg, Germany; 3 Department of General, Visceral and Transplantation Surgery, University of Heidelberg, Heidelberg, Germany

## Abstract

The repeated treatment of cancer cells with chemo- or radiotherapy induces therapy resistance, but it was previously unknown whether the same effect occurs upon continuous exposure of cancer cells to diet-derived chemopreventive agents. We elucidated this interesting question in pancreatic ductal adenocarcinoma, which is a highly aggressive cancer entity with a marked resistance toward gemcitabine and other cytotoxic drugs. The isothiocyanate sulforaphane, present in cruciferous vegetables, and the polyphenol quercetin, present in many fruits and vegetables induced apoptosis and reduced viability in gemcitabine-sensitive BxPC-3 cells but not in non-malignant ductal pancreas cells and mesenchymal stromal cells. In turn, BxPC-3 cells were treated with increasing concentrations of gemcitabine, sulforaphane or quercetin for more than 1 year and the surviving subclones Bx-GEM, Bx-SF and Bx-Q were selected, respectively. While Bx-GEM cells acquired a total resistance, Bx-SF or Bx-Q cells largely kept their sensitivity as proved by MTT assay, annexin staining and FACS analysis. The evaluation of the self-renewal-, differentiation- and migration-potential by colony formation, differentiation or migration assays demonstrated that cancer stem cell features were enriched in gemcitabine-resistant cells, but decreased in sulforaphane- and quercetin-long time-treated cells. These results were confirmed by orthotopic xenotransplantation of cancer cells to the mouse pancreas, where Bx-GEM formed large, Bx-Q small and Bx-SF cells almost undetectable tumors. An mRNA expression profiling array and subsequent gene set enrichment analysis and qRT-PCR confirmed that tumor progression markers were enriched in Bx-GEM, but reduced in Bx-SF and Bx-Q cells. This study demonstrates that the continuous exposure of pancreatic cancer cells to sulforaphane or quercetin does not induce resistance in surviving cells but reduces tumorigenicity by inhibition of tumor progression markers. These results highlight that cancer cells may not adapt to the preventive and therapeutic effects of a regular fruit- and vegetable-based diet.

Pancreatic ductal adenocarcinoma (PDA) is a highly aggressive malignancy, which is reflected by it's tenth place of estimated new cancer cases per year, but it's fourth place of estimated cancer deaths in males.^1^ Surgical resection is the only potentially curative therapy, but merely 15–20% of tumors are resectable, due to early metastasis, missing early symptoms and late diagnosis.^2^ Gemcitabine is considered as standard chemotherapy in PDA treatment, despite a low rate of responsiveness due to a marked resistance to chemo- and radiotherapy.^3^ The newer combination chemotherapy FOLFIRINOX extends life by 4 months when compared with gemcitabine but has more side effects.^4^

Chemoresistance, either acquired or intrinsic, is a major limitation in the successful treatment of pancreatic cancer. The frequent application of chemotherapy to cancer patients is due to the observation that it often succeeds in reducing a tumor mass and improves survival. However, the transition of the cancer to a resistant stage, called acquired resistance, is a key factor for the failure of chemotherapeutic agents.^5^ Recently, the high intrinsic resistance of pancreatic cancer was associated with a high basal percentage of the otherwise small amount of cancer stem cells (CSCs).^6^ Also, tumor progression was associated with the enrichment of CSCs, for example, of PDA,^7^ that survive anti-proliferative chemotherapeutics and contribute to disease progression.^8^

CSCs are considered to possess 'stemness' like normal stem cells including an enhanced tumor initiating potential, and the ability to tumorigenicity, self-renewal, differentiation and migration.^9, 10^ Various dysregulated signaling pathways have an important role in maintaining the stemness character of CSCs including self-renewal, epithelial–mesenchymal transition (EMT) and others.^11^ In solid tumors, chemotherapy-resistant CSCs were commonly detected, for example, in cancer of the breast,^12^ colorectum,^13^ prostate,^14^ ovary,^15^ lung,^16^ liver,^17^ glioblastoma,^18^ osteosarcoma^19^ and PDA.^20^ In particular, the enrichment of CSCs and drug resistance was found in PDA after repeated treatment with gemcitabine.^21^

Several epidemiological studies suggest that cancer development and progression are possibly correlated to a defined dietary pattern. Silverman *et al.*^22^ found in a large population-based case–control study that the consumption of cruciferous vegetables, for example, broccoli, cauliflower and cabbage, three and more times weekly, reduced the risk of pancreatic cancer by about 50% and therefore could have a preventive role. The main bioactive substance from broccoli and cauliflower is the mustard oil and isothiocyanate sulforaphane. Besides, the antioxidative polyphenol quercetin is present in broccoli and other cabbage varieties, but also in many other fruits and vegetables, such as berries, onions and apples.^23^ Several laboratory and animal studies exist and suggest that sulforaphane and quercetin inhibit proliferation and metastasis and enhance apoptosis and eliminate CSC features in pancreatic cancer. Therefore, these bioactive agents are considered as promising future treatment options.^24, 25, 26, 27, 28, 29^

The question is whether a future therapeutic treatment with sulforaphane or quercetin may induce drug resistance after frequent exposure, as known for chemotherapy. In the present study, we showed that frequently repeated cycles of sulforaphane and quercetin exposure did not induce drug resistance but reduced the tumorigenic potential and the expression of progression markers. In contrast, continuous exposure to gemcitabine induced a total drug resistance along with enhanced tumorigenicity.

## Results

### Quercetin and sulforaphane selectively reduce the viability in malignant cells

**Figure 1:**
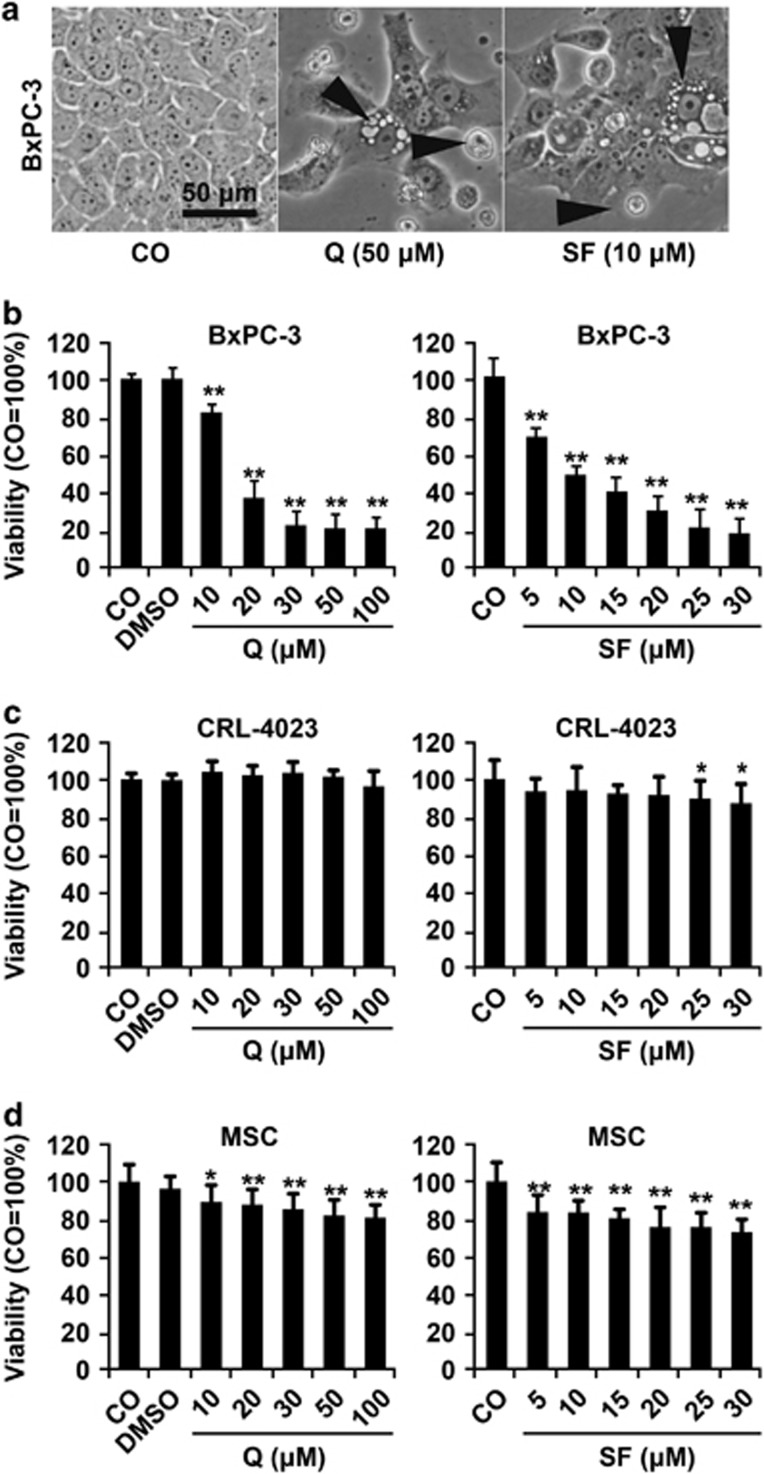
Quercetin and sulforaphane are selectively cytotoxic in pancreatic cancer cells. (**a**) The human PDA cell line BxPC-3 was left untreated (CO) or was treated with quercetin (Q, 50 *μ*M) or sulforaphane (SF, 10 *μ*M), followed by microscopy and photography 48 h later. Representative pictures at × 200 magnification are shown. (**b**) Cell viability was determined by MTT assay in untreated BxPC-3 cells (CO) or 72 h after treatment with vehicle alone diluted 1 : 1000 (DMSO) or with increasing concentrations of quercetin (Q) from 5 to 100 *μ*M and sulforaphane (SF) from 5 to 30 *μ*M, diluted in DMSO whose final concentration in medium was 1 : 1000 or higher. (**c**) The non-malignant primary human cell lines CRL-4023 (immortalized ductal pancreas cells) or (**d**) MSC (bone marrow-derived) were treated with different concentrations of quercetin (Q) or sulforaphane (SF) as indicated, while DMSO (1 : 1000) was set as a control. The cell viability was measured as described above. Three independent experiments were performed at least in triplicates and the data are presented as means±S.D. *P<0.05, **P<0.01

To establish the optimal dose response of PDA cell lines to quercetin and sulforaphane, the established gemcitabine-sensitive PDA cell line BxPC-3 was used, along with non-malignant primary human pancreatic duct cells (CRL-4023) and human mesenchymal stromal cells, which served as controls. After treatment with different concentrations of quercetin or sulforaphane, the cell morphology and viability were determined 72 h later by microscopy and MTT assay. The number of BxPC-3 cells strongly decreased after treatment and apoptotic blebbing appeared (Figure 1a). Quercetin and sulforaphane significantly inhibited the viability of BxPC-3 cells in a dose-dependent manner (Figure 1b). In contrast, different quercetin concentrations did not reduce the viability of the non-malignant cell line CRL-4023. Likewise, only high SF doses slightly affected the viability of CRL-4023 cells at 72 h. Similarly, quercetin or sulforaphane only weakly reduced the viability of mesenchymal stromal cells (Figure 1d). The DMSO solvent alone, at a dilution of 1 : 1000, which was the lowest concentration of dilution of the DMSO quercetin or sulforaphane stock in medium, did not affect the cell viability (Figures 1b–d), as expected.

### Selection of BxPC-3 subclones by continuous exposure to gemcitabine, sulforaphane or quercetin

**Figure 2:**
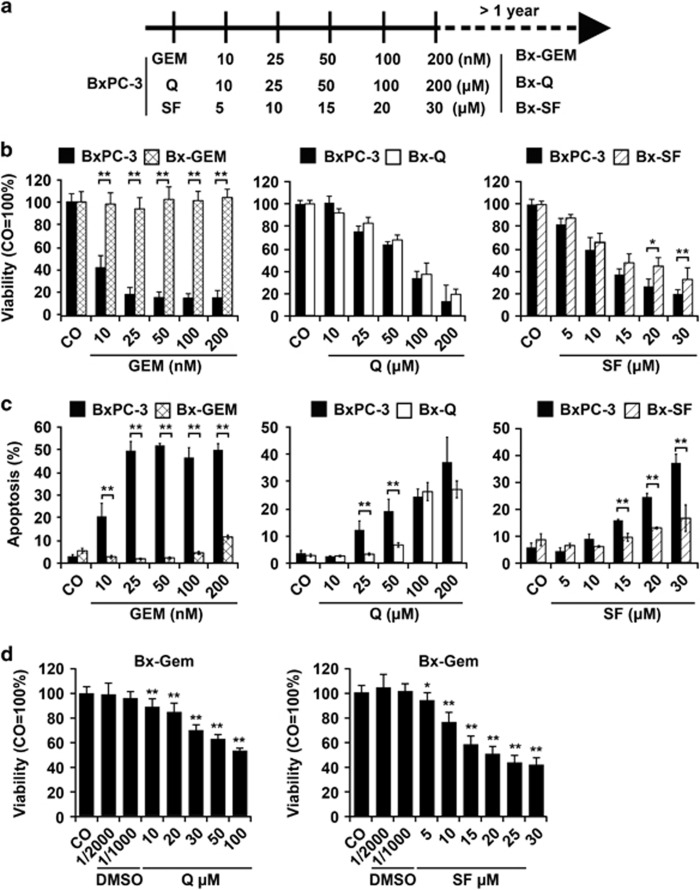
Continuous exposure to gemcitabine but not to sulforaphane or quercetin leads to pronounced therapy resistance. (**a**) BxPC-3 cells were treated with gemcitabine (GEM, 10 nM), quercetin (Q, 10 *μ*M) or sulforaphane (SF, 5 *μ*M) at a confluence of 40–60%. After 2 weeks, when the cells recovered, they were treated again with higher concentrations of each agent as indicated, followed by a recovery phase and a new round of treatment with higher concentrations. After more than 1 year of repeated treatment with gradually higher concentrations, the highest concentrations of 200 nM GEM, 200 *μ*M Q and 30 *μ*M SF were reached and the resulting final subclones were named Bx-GEM, Bx-Q and Bx-SF, respectively. These final subclones were used for all following experiments. (**b**) The cells were treated with different concentrations of gemcitabine, quercetin or sulforaphane as indicated. Seventy-two hours later, the viability was measured by MTT assay. (**c**) Likewise, the percentage of apoptotic cells was measured by staining with Annexin V-FITC and PI followed by FACS analysis. (**d**) Bx-GEM cells were treated with quercetin or sulforaphane at concentrations indicated; and 72 h later, the viability was measured by MTT assay. Three independent experiments were performed for (**b**–**d**) (MTT assay *n*=8, apoptosis assay *n*=3) and the data are presented as means ±S.D. *P<0.05, **P<0.01

To compare the effects of long-time treatment with gemcitabine, quercetin and sulforaphane, the K-Ras wildtype and gemcitabine-sensitive PDA cell line BxPC-3 was chosen to enable the selection of resistant subclones by continuous long-time treatment. Thus, BxPC-3 cells were exposed to continuously higher concentrations of gemcitabine, quercetin or sulforaphane for more than 1 year (Figure 2a). The final surviving subclones were selected and named Bx-GEM, Bx-Q and Bx-SF. To determine whether the subclones acquired resistance toward the respective treatments, they were left untreated for 2 weeks and then treated again. The percentages of viability and apoptosis were determined 72 h later by MTT assay (Figure 2b) or by staining with Annexin V-FITC and PI, followed by FACS analysis (Figure 2c). Although Bx-GEM cells were totally resistant to gemcitabine, even with the highest concentration of 200 nM, as we confirmed in our recent study,^30^ the treatment of Bx-Q cells with quercetin or of Bx-SF cells with sulforaphane still significantly reduced the viability and enhanced apoptosis. However, both Bx-Q and Bx-SF subclones were slightly more resistant to quercetin or sulforaphane, compared with parental BxPC-3 cells. Most importantly, totally gemcitabine-resistant Bx-GEM cells were sensitive to quercetin or sulforaphane, as concluded from a reduced viability after treatment (Figure 2d). To examine whether a change in the genetics of Bx-GEM cells might have been the reason for the gemcitabine resistance, we forwarded them together with parental BxPC-3 cells to a commercially available Multiplex Cell Line Authentication Test. In the resulting report, both BxPC-3 and Bx-GEM cells were confirmed to be 100% identical with the original BxPC-3 cells in the database (data not shown), suggesting that the resistance may be due to the upregulation of resistance mechanisms.

### Continuous quercetin and sulforaphane exposure reduces tumorigenicity *in vitro*

**Figure 3:**
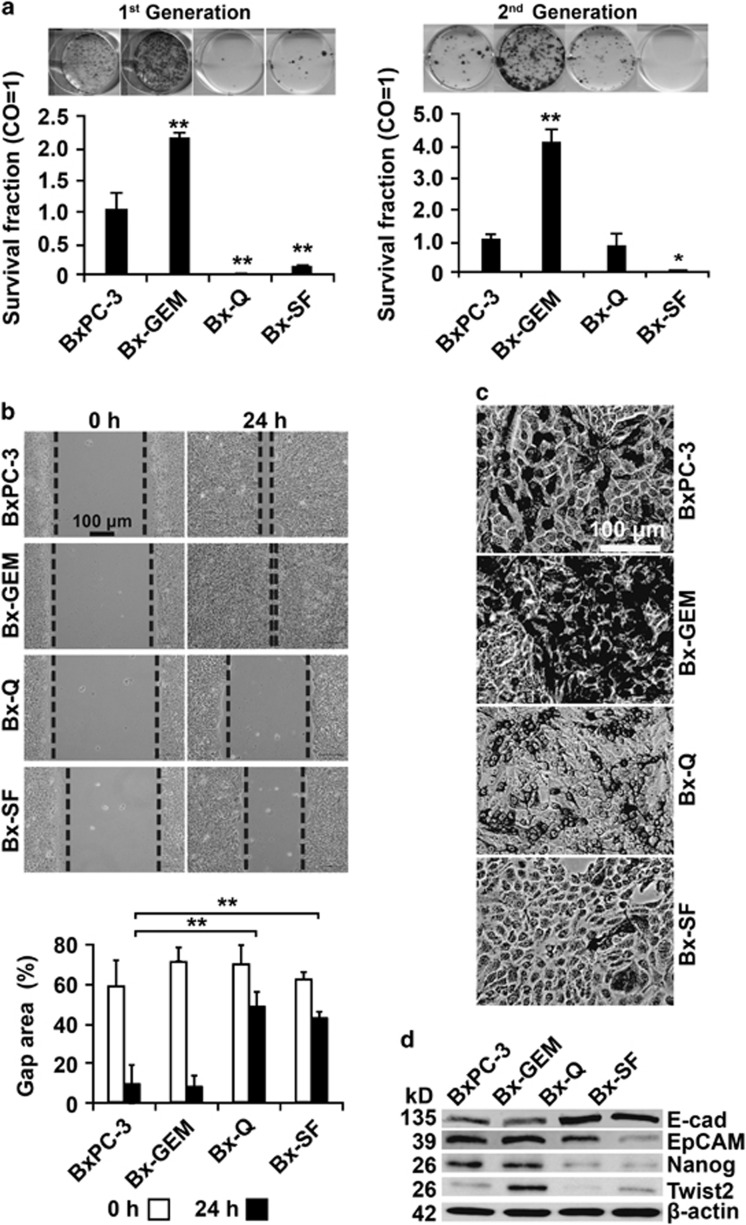
Continuous exposure to gemcitabine increases tumorigenicity but continuous exposure to sulforaphane or quercetin reduces it. (**a**) BxPC-3, Bx-GEM, Bx-Q and Bx-SF cells were seeded at a low density (2000 cells/well) in 6-well plates. After 2 weeks, cells were Coomassie-stained and colonies containing more than 50 cells were counted under a dissecting microscope. The survival fraction and representative photographs of colonies (first generation) are presented on the left. For second-generation colony formation, an equal amount of living cells from first-generation colonies were collected and 2000 cells per well were re-seeded. The colony formation was analyzed as described above and is presented on the right. (**b**) Cells were cultured to 90% confluence before the cell layer was scratched with the tip of a 10-*μ*l pipette. Closure of the wounded region was evaluated 24 h after scratching by microscopy at × 100 magnification. For quantification of the scratched area, the percentage of the gap area was evaluated and calculated by TScratch software (diagram below photographs). (**c**) Cells were seeded in 6-well plates, followed by exposure to NH Osteo-Diff medium to induce osteocytic differentiation. Fourteen days later, the cells were stained with BCIP/NBT substrate for alkaline phosphatases, expressed by cells differentiated into osteocytes, which appear dark. Representative images at × 200 magnification are shown. (**d**) Proteins were harvested and the expression of EpCAM, Nanog, Twist2 and E-cadherin was measured by western blot analysis. *β*-Actin was used as a loading control. Three independent experiments were performed at least in triplicates and the data are presented as means ±S.D. *P<0.05, **P<0.01

To investigate CSC features, we evaluated the colony formation, migration and differentiation capacities of parental BxPC-3 cells and their subclones. The cells were treated and 3 days later an equal number of surviving cells were seeded for colony formation. Ten days later, this resulted in a significantly enhanced number of colonies in Bx-GEM cells, but in a lower number in Bx-Q and Bx-SF cells compared with parental BxPC-3 cells (Figure 3a). To evaluate the long-lasting effect of treatment, an equal number of surviving cells from colonies of each group were selected, followed by re-seeding and detection of second-generation colonies 2 weeks later. While Bx-GEM cells still exhibited a high colony-forming capacity, Bx-Q and Bx-SF cells had a low to nearly absent colony-forming capacity. To elucidate the invasion potential, a scratch assay was performed. A confluent cell layer was wounded with the tip of a 10-*μ*l pipette; and the closure of the wounded region was evaluated by microscopy 24 h later. Whereas the gap was totally closed in Bx-GEM cells, it was still slightly open in BxPC-3 cells and widely open in Bx-Q and Bx-SF cells (Figure 3b). Then, the differentiation potential was tested by culturing the cells in osteogenic differentiation medium for 14 days, followed by staining with SIGMAFAST BCIP/NBT (Sigma-Aldrich, St. Louis, MO, USA) substrate for detection of alkaline phosphatase produced by cells differentiated into osteoblasts. We found that the Bx-GEM cells differentiated with a high potential, whereas the differentiation potential of Bx-Q and Bx-SF cells was reduced compared with parental BxPC-3 cells (Figure 3c). To further define cancer progression markers, we detected the expression of the CSCs surface marker EpCAM (also known as ESA), the self-renewal marker Nanog, the mesenchymal cell marker Twist2 and the epithelial cell marker E-cadherin by western blot analysis (Figure 3d). Whereas the expression of EpCAM, Nanog and Twist2 was almost reduced in Bx-Q and Bx-SF cells, E-cadherin was induced, but the opposite occurred in Bx-GEM cells, compared with parental BxPC-3 cells. Therefore, continuous exposure to gemcitabine strongly enhanced progression markers, whereas long-time treatment with quercetin or sulforaphane reduced them.

### Continuous quercetin and sulforaphane exposure reduces tumorigenicity *in vivo*

**Figure 4:**
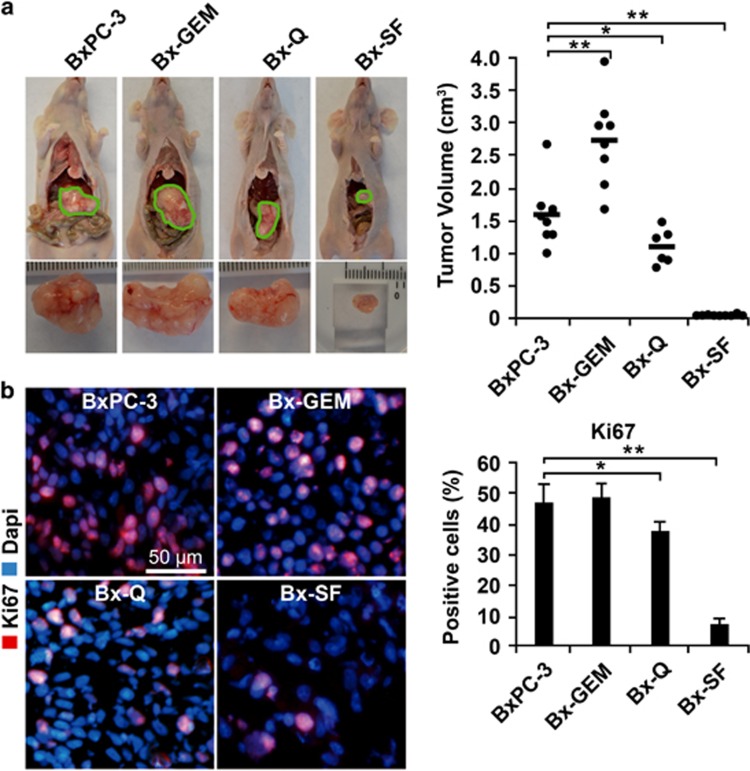
Continuous exposure to quercetin or sulforaphane inhibits tumorigenicity in *vivo*, whereas gemcitabine-treatment enhances it. (**a**) Immunodeficient mice (*n*=8/per cell line) were anesthetized, followed by surgical intervention to expose the pancreas and to inject 1 × 10^5^ cells in 10 *μ*l matrigel into the pancreatic head. After closure of the wound, the mice were kept for 6 weeks to allow tumor development, followed by euthanasia, tumor resection and measurement of tumor volumes by calipers. Representative images of tumor xenografts from each group are shown on the left and a diagram with the individual tumor volumes and the means of each group ±S.D. on the right. (**b**) Tumor tissue sections from xenografts were evaluated by immunofluorescence staining for the expression of the proliferation marker Ki67 and representative pictures at × 200 magnification are shown on the left. The percentage of positive cells was counted and the means ±S.D. are shown in the diagram on the left. *P<0.05, **P<0.01

To further compare the tumorigenicity between parental BxPC-3 cells and the derived subclones *in vivo*, an equal number of each cell line was orthotopically transplanted to the pancreas of immunodeficient mice. Six weeks later, the mice were killed and the xenograft tumors resected, followed by the measurement of the tumor volumes. Compared with the parental cells, the tumor volume significantly increased in mice harboring Bx-GEM xenografts, but it was reduced in mice with Bx-Q tumors and almost not detectable in mice with Bx-SF xenografts (Figure 4a). To evaluate the expression of proliferation marker Ki67, we performed immunofluorescence staining followed by microscopy (Figure 4b). Whereas Bx-GEM cells had a slightly higher, but not significantly different proliferation rate compared with BxPC-3 cells, the proliferation was significantly decreased in Bx-Q xenografts and further diminished in tumors derived from Bx-SF cells. Because the expression of the proliferation marker Ki67 did not significantly differ between xenografts of parental BxPC-3 and Bx-GEM cells, we wondered whether the larger tumor volume of Bx-GEM cells might be due to a reduced basal apoptosis. Thus, we stained the cleaved fragment of active caspase-3 in xenograft tissue and evaluated the percentage of positive cells in 10 vision fields. Though the percentage of apoptosis was lower in Bx-GEM-derived xenografts compared with BxPC-3-derived tumors, this difference was not statistically significant (Supplementary Figure 1).

### Continuous quercetin and sulforaphane exposure reduces gene array-analyzed expression of progression markers

**Figure 5:**
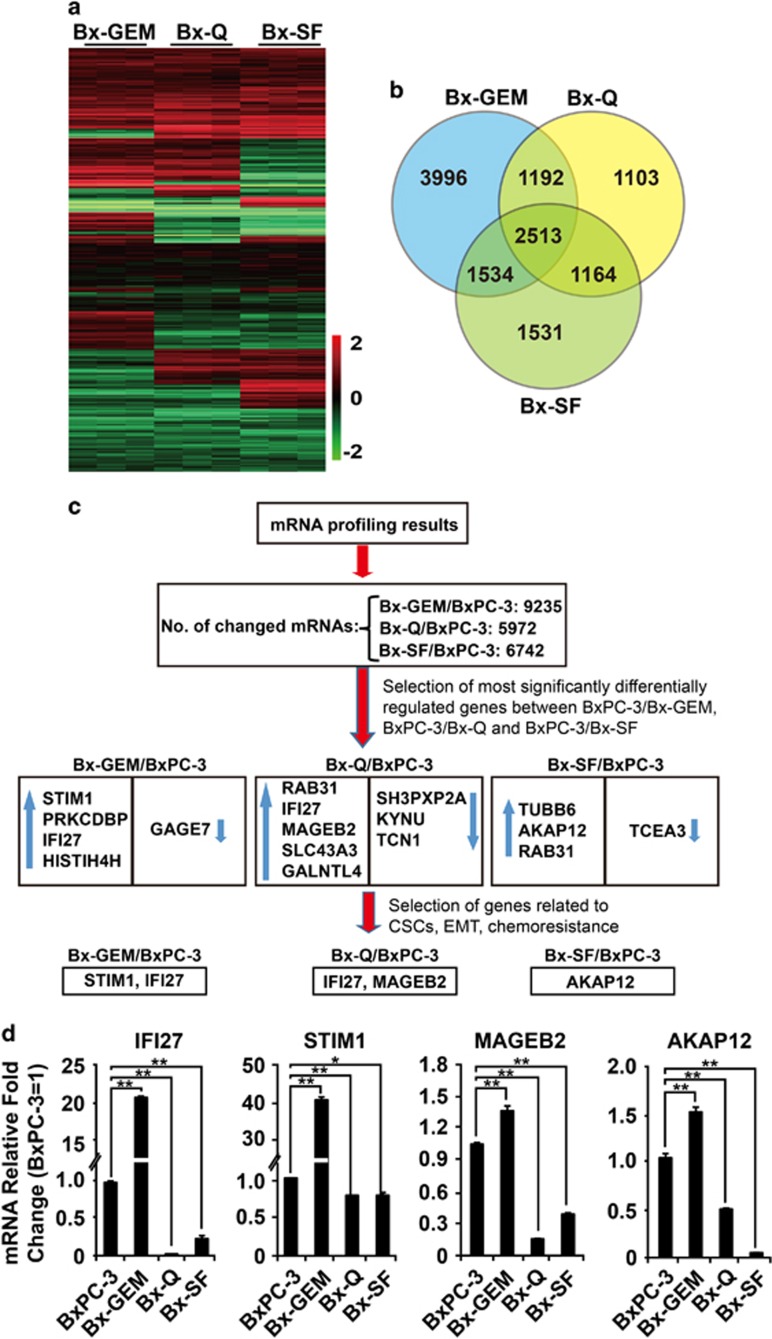
Gene array analysis demonstrates differentially regulated genes after continuous exposure to sulforaphane, quercetin or gemcitabine. (**a**) mRNA was harvested from BxPC-3, Bx-GEM, Bx-Q and Bx-SF cells followed by gene array analysis and biostatistical evaluation of statistically significant expression patterns between the different cell lines. The heat map shows the relative expression of differentially regulated genes in Bx-GEM, Bx-Q and Bx-SF cells compared with parental BxPC-3 cells. (**b**) The numbers of differentially regulated genes in Bx-GEM, Bx-Q and Bx-SF cells compared with parental BxPC-3 cells and compared between the groups are presented in a Venn diagram (*P*<0.01). (**c**) Flow diagram of the computational selection process of miRNA candidates from the miRNA profiling results is shown above. Upward blue arrows: upregulation of mRNAs compared with BxPC-3; Downward blue arrows: downregulation of mRNAs compared with BxPC-3. (**d**) RNA was harvested from BxPC-3, Bx-GEM, Bx-Q and Bx-SF cells; and mRNA expression of *IFI27*, *STIM1*, *MAGEB2* and *AKAP12* was analyzed by qRT-PCR. The expression in BxPC-3 cells was set to 1. GAPDH was used as an endogenous control. The qRT-PCR was performed in triplicates three times with similar outcome; and the means ±S.D. are shown

To investigate CSC features on the mRNA expression level, total RNA from parental BxPC-3 cells and the derived final subclones Bx-GEM, Bx-Q and Bx-SF was extracted; and an mRNA profiling analysis was performed from triplicates by the use of the Human HT-12 v4 Expression Bead Chip Kit with 44 052 genes. The relative expression of significantly changed genes (*P*<0.01) is shown in a heat map (Figure 5a). The number of differentially expressed mRNAs in parental cells compared with the derived three subclones is presented as a Venn diagram (Figure 5b). A comparison of gene expression between Bx-GEM, Bx-Q and Bx-SF cells revealed the differential regulation of 2513 genes. Among them, 1192 genes were differentially regulated between Bx-GEM and Bx-Q cells, 1534 between Bx-GEM and Bx-SF cells and 1164 genes between Bx-Q and Bx-SF cells. Compared with parental BxPC-3 cells, 9235, 5972 and 6742 genes were differentially regulated in Bx-GEM, Bx-Q and Bx-SF cells, respectively (Figure 5c). Next, we selected those candidate genes, which were most significantly differentially regulated and related to CSCs, by chemoresistance or EMT by the use of the Ingenuity Pathway Analysis (IPA) computer program. The expression patterns of the predicted candidate genes were confirmed by qRT-PCR (Figure 5d). Interferon alpha-inducible protein 27 (*IFI27)*, which confers EMT, tumorigenicity and stemness,^31^ stromal interaction molecule 1 (*STIM1*), which promotes tumor metastasis^32^ and apoptosis resistance,^33^ melanoma antigen family B2 (*MAGEB2*), which activates cell proliferation and resistance to ribotoxic stress,^34^ and kinase anchor protein 12 (*AKAP12)*, which promotes tumor development and metastasis,^35^ were all upregulated in Bx-GEM cells but downregulated in Bx-Q and Bx-SF cells compared with parental BxPC-3 cells.

### Continuous quercetin and sulforaphane exposure reduces the expression of progression markers

**Figure 6:**
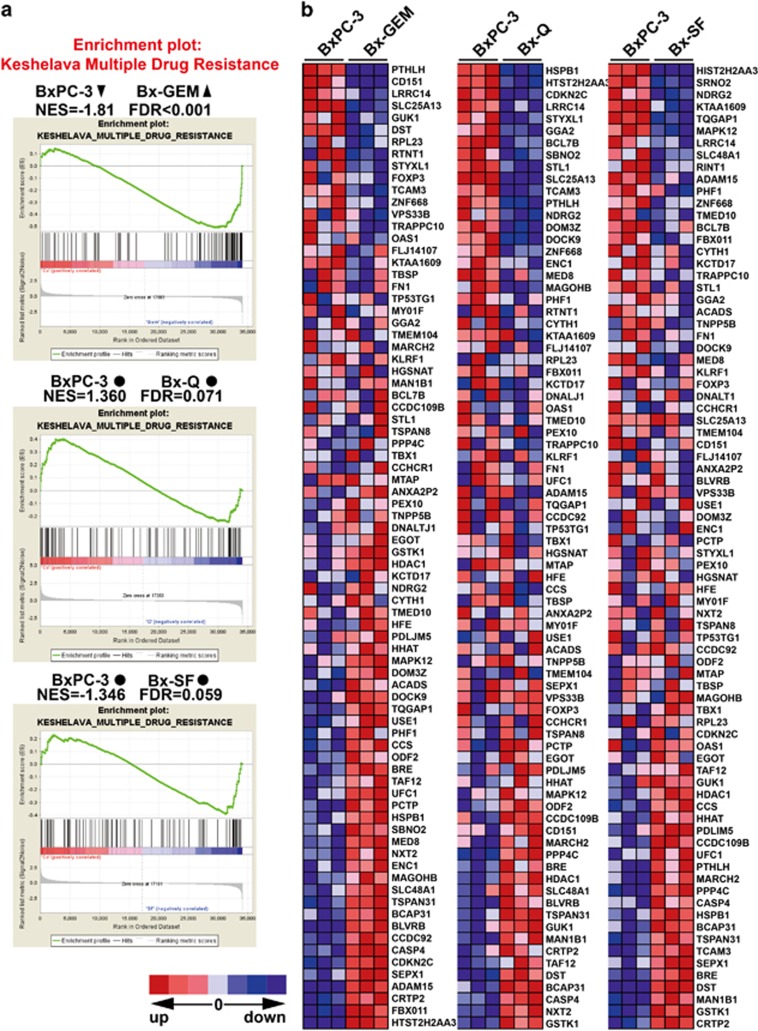
Gene set enrichment analysis demonstrates the induction of multidrug resistance in Bx-GEM but not in Bx-Q and Bx-SF cells. (**a**) Significantly differentially regulated genes from the gene array analysis described in Figure 5 were further analyzed by a gene set enrichment analysis with an available set of genes known to be involved in multidrug resistance (KESHELAVA_MULTIPLE_DRUG_RESISTANCE). NES, normalized enrichment score; FDR, false discovery rate. ▴ Enriched. **●** No change. **▾** Decreased. The differences were considered significant if FDR values were less than 0.05. The detailed process is described in Materials and Methods. (**b**) Heat map of the differentially regulated mRNAs according to gene set enrichment analysis as described in (**a**). The scale marks the relative changes of differentially regulated mRNAs in BxPC-3, Bx-GEM, Bx-Q and Bx-SF: upregulated (red), no change (gray) and downregulated (blue)

**Figure 7:**
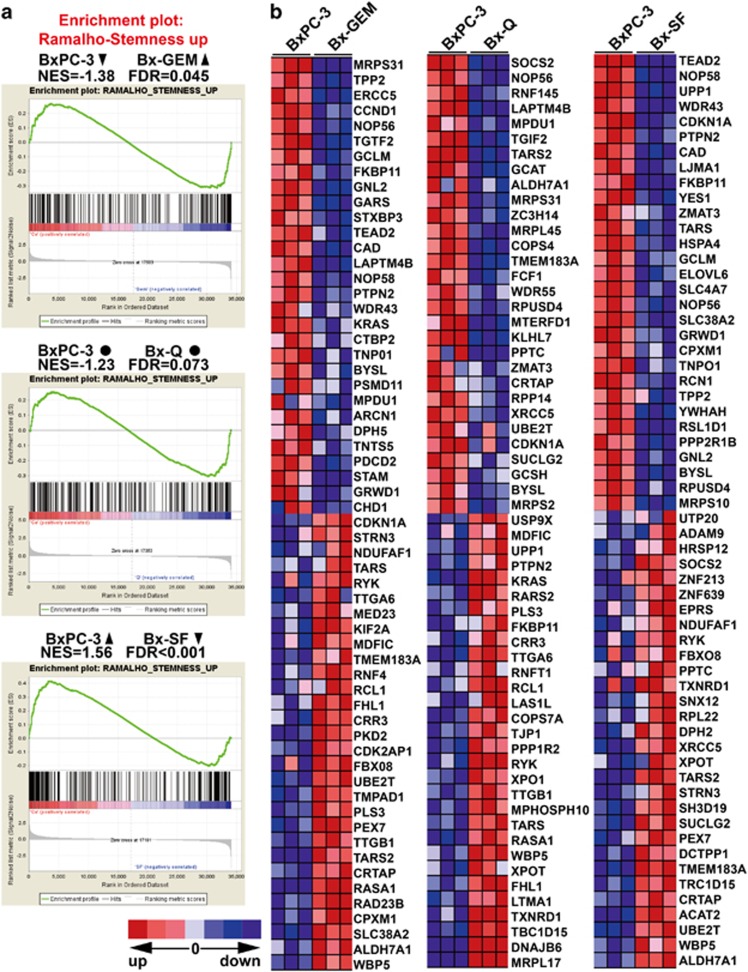
Gene set enrichment analysis demonstrates the induction of stemness genes in Bx-GEM but reduction in Bx-SF and no change in Bx-Q cells. (**a**) Significantly differentially regulated genes from the gene array analysis described in Figure 5 were further analyzed by a gene set enrichment analysis with an available set of genes known to be involved in stemness (RAMALHO_STEMNESS_UP) and further analyzed as described in Figure 6a. (**b**) Heat map of the differentially regulated mRNAs according to gene set enrichment analysis as described in (**a**)

To characterize the gene array results by an additional computational method, we performed a gene set enrichment analysis (GSEA) to identify those differentially regulated genes typical for drug resistance and stemness. The GSEA computational method determines whether an *a priori* defined set of genes shows statistically significant, concordant differences between two biological states (http://www.broadinstitute.org/gsea/index.jsp), or in our case, between parental BxPC-3 cells and the derived subclones Bx-GEM, Bx-Q or Bx-SF. We used the ready-to-use KESHELAVA_MULTIPLE_DRUG_RESISTANCE set, which includes 88 genes related to chemoresistance and the RAMALHO_STEMNESS_UP set, which includes 206 genes, known to be enriched in embryonic, neural and hematopoietic stem cells (compare Supplementary Table 1).^21^ Regarding the expression of multidrug-resistance genes, Bx-Q and Bx-SF cells showed no significant changes compared with parental BxPC-3 cells, but Bx-GEM cells had a significant upregulation (Figure 6a). The detailed differential expression of each gene is shown in the heat map (Figure 6b). For instance, FBX011, which served as an oncogene in breast cancer and was related to chemoresistance,^36^ was significantly upregulated in Bx-GEM cells compared with BxPC-3 cells. Likewise, Bx-GEM cells exhibited a significant enrichment of genes related to stemness features, whereas Bx-SF cells had a significantly decreased expression compared with parental BxPC-3 cells and no significant change was found in Bx-Q cells (Figures 7a and b). For example, compared with BxPC-3, Bx-SF exhibited a lower level expression of the YES-YAP-TEAD2 signaling pathway, which was reported to maintain embryonic stem cell self-renewal.^37^

## Discussion

In the present study, the situation of acquired therapy resistance was mimicked by long-time treatment of gemcitabine-sensitive BxPC-3 cells with continuously increasing concentrations of gemcitabine for more than 1 year, to select the drug-resistant, final population, as described.^38^ In addition, we investigated the important question, whether pancreatic cancer can acquire resistance by the continuous exposure to sulforaphane or quercetin, resembling a long-time fruit- and vegetable-enriched diet. Whereas the gemcitabine-selected clone Bx-GEM was totally resistant toward gemcitabine, it was still sensitive to quercetin and sulforaphane. Likewise, long-time quercetin- or sulforaphane-treated Bx-Q or Bx-SF cells were only slightly resistant and almost kept their sensitivity toward the respective stimulus. Although most of the long-time sulforaphane- or quercetin-treated BxPC-3 cells underwent apoptosis after each new round of sulforaphane or quercetin treatment, a small percentage of cells survived. The reason for this slight adaption to these bioactive agents is unclear, but we found that it is not due to the activation of conventional resistance mechanisms. Accordingly, Bx-Q and Bx-SF cells had a lower colony- and spheroid-forming capacity and migrated slower compared with parental BxPC-3 cells. In contrast, these tumorigenic features were enhanced in Bx-GEM. Our data suggest that an enhanced self-renewal potential was rather not the reason for the observed slight apoptosis resistance of Bx-Q cells to quercetin and of Bx-SF cells to sulforaphane. In support of this hypothesis, the examination of the differentiation potential and the protein expression of EMT and stemness markers revealed an enhanced differentiation potential in Bx-GEM, but a reduced potential in Bx-SF and Bx-Q cells. Therefore, we assume that Bx-Q and Bx-SF cells adapted somehow to quercetin or sulforaphane treatment, although this adaption is not due to enhanced tumorigenicity and the underlying mechanism requires further investigation.

These *in vitro* data were further underlined by orthotopic xenotransplantation to the mouse pancreas, which resulted in a significantly reduced tumor volume after transplantation of Bx-SF or BX-Q cells, whereas Bx-GEM cells formed much larger tumors compared with parental BxPC-3 cells. In correspondence to the colony-forming potential *in vitro*, tumors derived from Bx-SF cells even smaller or totally absent than those derived from Bx-Q cells. Interestingly, although the means of Ki67-positive cells in tumor tissue were higher in Bx-GEM and lower in Bx-Q and Bx-SF cells compared with BxPC-3 cells, and *vice versa* for the cleaved fragment of active caspase-3, these differences were not statistically relevant. Therefore, we assume that the observed higher tumor volume of Bx-GEM-derived xenografts may be due to a slightly higher proliferation rate and a slightly lower basal apoptosis rate, which cannot be proved by the rather insensitive method of immunofluorescence staining and counting the number of positive cells by visual inspection.

To get knowledge about differential gene expression, which might be responsible for the observed effects, we performed mRNA profiling, selected differentially regulated candidate genes linked to EMT, CSC and chemoresistance signaling and confirmed their expression by qRT-PCR. In addition, a GSEA was used to compare the gene difference between parental cells and the derived subclones. Although the candidates identified by both selection methods were not identical, we demonstrated that multidrug resistance- and stemness-associated genes were enriched in Bx-GEM cells but not in Bx-Q and Bx-SF cells. Most excitingly, stemness-related genes were even decreased in Bx-SF cells.

In the clinical settings, it is important to achieve a therapeutically sufficient plasma concentration of quercetin or sulforaphane. The plasma concentration typically found in humans after consumption of foods containing flavonoids is between 0.06 and 7.6 *μ*M, which corresponds to recently detected plasma concentrations of quercetin.^39^ Peak plasma concentrations of 108.7±41.67 *μ*M quercetin have been observed;^40^ and a tolerance of quercetin doses ranging from 60 mg/m^2^ (=1.5 mg/kg) to 1700 mg/m^2^ (=42.5 mg/kg) is known.^41^ However, the therapeutic active doses of quercetin are difficult to be realized by nutrition alone. For example, to achieve about 500 mg of quercetin, this would amount to the consumption of 1 L of red wine (19 mg), 1 kg apples (140 mg), 1 kg yellow onions (347 mg) or 1 kg broccoli (30 mg).^27^ Regarding sulforaphane intake, data from a prospective Canadian epidemiological study suggest that a high consumption of broccoli or cauliflower at almost one serving per week, while three to five servings were more effective, is associated with inhibition of metastasis in prostate cancer.^42^ Experimental sulforaphane concentrations, which inhibited growth of human pancreatic cancer xenografts on mice, were 4.4 mg/kg per day.^24^ Extrapolating this experimental concentration to humans suggests a dose of 0.36 mg/kg according to the body surface area normalization method.^43^ This corresponds to 25 mg/70 kg human, which is hard to be reached by eating mature broccoli florets alone. However, an alternative to consumption of mature broccoli for high intake of sulforaphane may be the intake of special freeze-dried broccoli sprouts or broccoli seed extract preparations. Such sprouts, seeds and derived extracts are available from several manufacturers, and they usually contain about 10–100 times more glucoraphanin, on a weight basis, than mature broccoli florets.^44^ Therefore, to achieve an effective concentration, supplements with high concentrations of quercetin or sulforaphane may improve the clinical outcome of chemotherapy.

In conclusion, our results highlight that a frequent consumption of quercetin or sulforaphane supplements may have preventive and therapeutic effects in pancreatic cancer.

## Materials and Methods

### Human primary and established cell lines

The human established pancreatic cancer cell line BxPC-3 and the human hTERT-HPNE immortalized pancreatic ductal cell line CRL-4023 were obtained from ATCC and cultured in DMEM supplemented with 10% FCS and 5% HEPES or in ATCC complete growth medium, respectively. Mesenchymal stromal/stem cells (MSCs) were isolated from bone marrow and cultured as described.^45^ All cells were grown in a humidified incubator at 37 °C and 5% CO_2_. BxPC-3 cells were recently authenticated by a commercial service (Multiplexion, Heidelberg, Germany). Mycoplasma-negative cultures were ensured by monthly mycoplasma tests.

### Reagents

Gemcitabine solution (Eli Lilly, Indianapolis, IN, USA) was obtained from the Pharmacy of the University Clinic of Heidelberg at a concentration of 76 mg/ml and stored at 4 °C. This stock was freshly diluted in DMEM to a 100-*μ*M stock. Quercetin (≥95%) and sulforaphane (≥95%) (Sigma-Aldrich, St. Louis, MO, USA) were dissolved in DMSO to stock concentrations of 200 mM or 100 mM, respectively, and stored in aliquots at −20 °C. Each stock was used only once immediately after thawing. The final concentrations of the solvents in media were 0.1% or less.

### Viability assay

The viability was measured using 3-(4,5-dimethylthiazol-2-yl)-2, 5-diphenyltetrazolium bromide (MTT) as described.^24^

### Measurement of apoptosis

The cells were stained with FITC-conjugated Annexin V (BD Biosciences, Heidelberg, Germany) and propidium iodide (5 mg/ml) after respective treatments and analyzed by flow cytometry (Guava EasyCyte, Cytometer, Millipore, Darmstadt, Germany), as described.^24^

### Colony formation assay

Cells were seeded at a low density in complete medium in 6-well tissue culture plates. Colonies were evaluated 10–14 days later as described.^24^

### Differentiation assay

Cells were seeded in 6-well plates. After 24 h, the medium was replaced by 2 ml osteogenic differentiation medium. The cells were cultured for 10 days. Differentiated cells were detected and images of stained cells were taken as described.^46^

### Wound healing assay

Cells (5 × 10^5^) were seeded in 6-well plates and grown to confluence. A line was then scraped within the confluent cells using a 10-*μ*l pipette tip. Images of the wound healing area were acquired by microscopy 24 h later as described; and the area was quantified by TScratch software as described.^47^

### Western blot analysis

Protein extracts were prepared by standard protocols and proteins were detected by western blot analysis as described.^24^ Antibodies were mouse monoclonal anti-EpCAM (HEA125),^48^ anti-Twist2 (Abcam, Cambridge, UK), anti-Nanog and anti-*β*-actin (Sigma-Aldrich) and rabbit monoclonal anti-E-cadherin (24E10, Cell Signalling Technology, Danvers, MA, USA).

### Immunofluorescence staining

Immunofluorescence staining of samples from tumor xenografts was performed as described.^24^ Mouse monoclonal antibodies against Ki67 (Abcam) were used.

### mRNA profiling

The RNeasy Mini Kit was used to extract total RNA according to the instructions of the manufacturer (Qiagen, Hilden, Germany); and the expression profiling was performed at the Genomics and Proteomics Core Facility of the German Cancer Research Center (DKFZ) Heidelberg, using the Human HT-12 v4 Expression Bead Chip Kit.

### qRT-PCR

The RNA concentrations were measured with a NanoDrop 2000 Spectrophotometer (Nano Drop Technologies, Wilmington, DE, USA); and 500 ng total RNA was reverse transcribed to cDNA using the High Capacity RNA to cDNA Kit (Thermo Fisher Scientific GmbH, Dreieich, Germany). Real-time PCR was performed using the Taqman Gene Expression master mix (Thermo Fisher Scientific). Primers for IFI27, STIM1, AKAP12, MAGEB2 and GAPDH were obtained from Thermo Fisher Scientific.

### Bioinformatics

GSEA was performed using the GSEA version 2.2.1 from the Broad Institute at MIT. The gene sets of KESHELAVA_MULTIPLE_DRUG_RESISTANCE and RAMALHO_STEMNESS_up were used. With 1000 permutations, the cutoff of the significance level was chosen as *P*<0.05. The GSEA is described in detail at http://software.broadinstitute.org/gsea/doc/GSEAUserGuideFrame.html.

### Nude mice and xenografts

A total of 1 × 10^5^ cells in 10 *μ*l matrigel were injected into the subscapular region near to the head of the pancreas of 6-week-old NMRI (nu/nu) male mice (day 0) through an abdominal midline incision with a fine needle after the mouse was under general anesthesia. To avoid possible leakage of tumor cells, the injection was performed under a Leica M651 microscope (Leica, Wetzlar, Germany). The mice were killed 6 weeks after tumor transplantation and the tumor volumes (*V*) were measured by two diameters and calculated using the formula *V*=1/2(length × width^2^). Animal experiments were performed in the animal facilities of the University of Heidelberg after receiving approval from the authorities (Regierungspräsidium Karlsruhe, Germany).

### Statistical evaluations

Data obtained with established cell lines are presented as the means ±S.D. from at least three separate experiments, each performed at least in triplicate. The mouse experiment was performed twice with statistically sufficient group sizes. The significance of the data was analyzed with Student's *t*-test for parametric data and the Mann–Whitney test with Bonferroni corrections for non-parametric data. *P*<0.05 was considered as statistically significant (**P<0.01, *P<0.05).

## Abbreviations

CSCs: cancer stem cells
PDA: pancreatic ductal adenocarcinoma
