# Supplementary material for: Continuous exposure of pancreatic cancer cells to dietary bioactive agents does not induce drug resistance unlike chemotherapy
Source: Cell Death Dis. 2016 Jun 2;7(6):e2246–. doi: 10.1038/cddis.2016.157 (PMC5143386; doi:10.1038/cddis.2016.157)
Supplement: Supplementary Table 1 [file cddis2016157x1.doc]

Supplemental Table 1 Names of genes of gene enrichment sets used in Fig. 6, 7

| KESHELAVA_MULTIPLE_DRUG_RESISTANCE set | | | | | | | |
| --- | --- | --- | --- | --- | --- | --- | --- |
| 1 | PTHLH | 21 | MYO1F | 41 | GSTK1 | 61 | UFC1 |
| 2 | CD151 | 22 | GGA2 | 42 | HDAC1 | 62 | PCTP |
| 3 | LRRC14 | 23 | TMEM104 | 43 | KCTD17 | 63 | HSPB1 |
| 4 | SLC25A13 | 24 | MARCH2 | 44 | NDRG2 | 64 | SBNO2 |
| 5 | GUK1 | 25 | KLRF1 | 45 | CYTH1 | 65 | MED8 |
| 6 | DST | 26 | HGSNAT | 46 | TMED10 | 66 | NXT2 |
| 7 | RPL23 | 27 | MAN1B1 | 47 | HFE | 67 | ENC1 |
| 8 | RINT1 | 28 | BCL7B | 48 | PDLIM5 | 68 | MAGOHB |
| 9 | STYXL1 | 29 | CCDC109B | 49 | HHAT | 69 | SLC48A1 |
| 10 | FOXP3 | 30 | SIL1 | 50 | MAPK12 | 70 | TSPAN31 |
| 11 | ICAM3 | 31 | TSPAN8 | 51 | DOM3Z | 71 | BCAP31 |
| 12 | ZNF668 | 32 | PPP4C | 52 | ACADS | 72 | BLVRB |
| 13 | VPS33B | 33 | TBX1 | 53 | DOCK9 | 73 | CCDC92 |
| 14 | TRAPPC10 | 34 | CCHCR1 | 54 | IQGAP1 | 74 | CASP4 |
| 15 | OAS1 | 35 | MTAP | 55 | USE1 | 75 | CDKN2C |
| 16 | FLJ14107 | 36 | ANXA2P2 | 56 | PHF1 | 76 | SEPX1 |
| 17 | KIAA1609 | 37 | PEX10 | 57 | CCS | 77 | ADAM15 |
| 18 | IBSP | 38 | INPP5B | 58 | ODF2 | 78 | CRIP2 |
| 19 | FN1 | 39 | DNALI1 | 59 | BRE | 79 | FBXO11 |
| 20 | TP53TG1 | 40 | EGOT | 60 | TAF12 | 80 | HIST2H2AA3 |
| RAMALHO_STEMNESS_UP set | | | | | | | |
| 1 | YES1 | 42 | C19orf2 | 83 | LRRC58 | 124 | ZNF281 |
| 2 | UPP1 | 43 | USP9X | 84 | KLHL7 | 125 | XPO1 |
| 3 | MRPL17 | 44 | ZZZ3 | 85 | C12orf45 | 126 | CCND1 |
| 4 | PPIC | 45 | SH3D19 | 86 | GAB1 | 127 | SNRPC |
| 5 | ITGB1 | 46 | CRTAP | 87 | RCN1 | 128 | RAB18 |
| 6 | MDFI | 47 | GRWD1 | 88 | DICER1 | 129 | GAS2 |
| 7 | WBP5 | 48 | SEC23IP | 89 | CTBP2 | 130 | XPOT |
| 8 | EIF4G2 | 49 | FBXO38 | 90 | CBR3 | 131 | BACH1 |
| 9 | LAPTM4B | 50 | C5orf51 | 91 | UMPS | 132 | SLC38A2 |
| 10 | EIF4EBP1 | 51 | PPP2R1B | 92 | NDUFAB1 | 133 | PLS3 |
| 11 | STRN3 | 52 | MPHOSPH10 | 93 | CAD | 134 | ARCN1 |
| 12 | MRPL45 | 53 | TOM1L1 | 94 | DDX1 | 135 | LIMA1 |
| 13 | LSG1 | 54 | PAFAH2 | 95 | INTS5 | 136 | PSMD11 |
| 14 | DPH5 | 55 | ZMYM4 | 96 | NDUFAF1 | 137 | GNB1 |
| 15 | WDR55 | 56 | RARS2 | 97 | TPP2 | 138 | CDKN1A |
| 16 | PKD2 | 57 | PACRGL | 98 | HSPA4 | 139 | FBXO8 |
| 17 | UBE2T | 58 | UTP20 | 99 | MSH2 | 140 | TRIP6 |
| 18 | RNF138 | 59 | PHTF2 | 100 | NOP56 | 141 | LAS1L |
| 19 | ACAT2 | 60 | SMAD2 | 101 | UBE2D2 | 142 | NUP35 |
| 20 | PRPF6 | 61 | ANKRD17 | 102 | ZMAT3 | 143 | RABGGTB |
| 21 | LSM2 | 62 | PEX7 | 103 | C6orf26 | 144 | IMPAD1 |
| 22 | ZNF213 | 63 | SEC23A | 104 | STAM | 145 | F2R |
| 23 | CHD1 | 64 | ZNF644 | 105 | NOP58 | 146 | PRPSAP1 |
| 24 | GNL2 | 65 | RPUSD4 | 106 | ACADM | 147 | ITGA6 |
| 25 | MPDU1 | 66 | BYSL | 107 | STXBP3 | 148 | ELP2 |
| 26 | ZFX | 67 | GFER | 108 | RPL22 | 149 | ARIH1 |
| 27 | COPS4 | 68 | RSL1D1 | 109 | COPS7A | 150 | RSRC2 |
| 28 | PTPN2 | 69 | PPA1 | 110 | MTMR10 | 151 | MED23 |
| 29 | TARS2 | 70 | GCLM | 111 | GLO1 | 152 | MAP4K3 |
| 30 | CIRH1A | 71 | MRPL34 | 112 | GARS | 153 | PPP1R2 |
| 31 | SNX12 | 72 | GCSH | 113 | TMEM183A | 154 | MRPL3 |
| 32 | KCNAB3 | 73 | PDCD2 | 114 | MKI67IP | 155 | HRSP12 |
| 33 | MDFIC | 74 | MRPS2 | 115 | SLC4A7 | 156 | XRCC5 |
| 34 | ZNF639 | 75 | MTERFD1 | 116 | JAGN1 | 157 | GSTA4 |
| 35 | SMAD1 | 76 | SUCLG2 | 117 | TGIF2 | 158 | RAD23B |
| 36 | ESF1 | 77 | PIGX | 118 | FKBP9 | 159 | RNF145 |
| 37 | MRPS31 | 78 | RYK | 119 | IARS | 160 | DNAJB6 |
| 38 | TBC1D15 | 79 | RPP14 | 120 | RNF4 | 161 | ZCCHC10 |
| 39 | ERCC5 | 80 | RNFT1 | 121 | TGS1 | 162 | CWC22 |
| 40 | CPXM1 | 81 | YAP1 | 122 | PSMD12 | 163 | DCTPP1 |
| 41 | ELOVL6 | 82 | GCAT | 123 | CTTN | 164 | EPRS |
| 165 | ADAM9 | 176 | FHL1 | 187 | CENPC1 | 198 | SLC7A6 |
| 166 | C17orf79 | 177 | YWHAH | 188 | FCF1 | 199 | PCF11 |
| 167 | RRN3 | 178 | TXNDC9 | 189 | RCL1 | 200 | TJP1 |
| 168 | KANK3 | 179 | FKBP11 | 190 | TBRG1 | 201 | KIF2A |
| 169 | LAPTM4A | 180 | PLA2G6 | 191 | WDR43 | 202 | TXNRD1 |
| 170 | TEAD2 | 181 | KRAS | 192 | SMARCAD1 |  |  |
| 171 | EIF3J | 182 | YWHAB | 193 | GHR |  |  |
| 172 | MRPS10 | 183 | BLZF1 | 194 | TNPO1 |  |  |
| 173 | ZC3H14 | 184 | ROCK2 | 195 | RASA1 |  |  |
| 174 | IARS2 | 185 | CDK2AP1 | 196 | SOCS2 |  |  |
| 175 | ALDH7A1 | 186 | DTYMK | 197 | USP10 |  |  |
