# Supplementary figures and images for: Continuous exposure of pancreatic cancer cells to dietary bioactive agents does not induce drug resistance unlike chemotherapy
Source: Cell Death Dis. 2016 Jun 2;7(6):e2246–. doi: 10.1038/cddis.2016.157 (PMC5143386; doi:10.1038/cddis.2016.157)

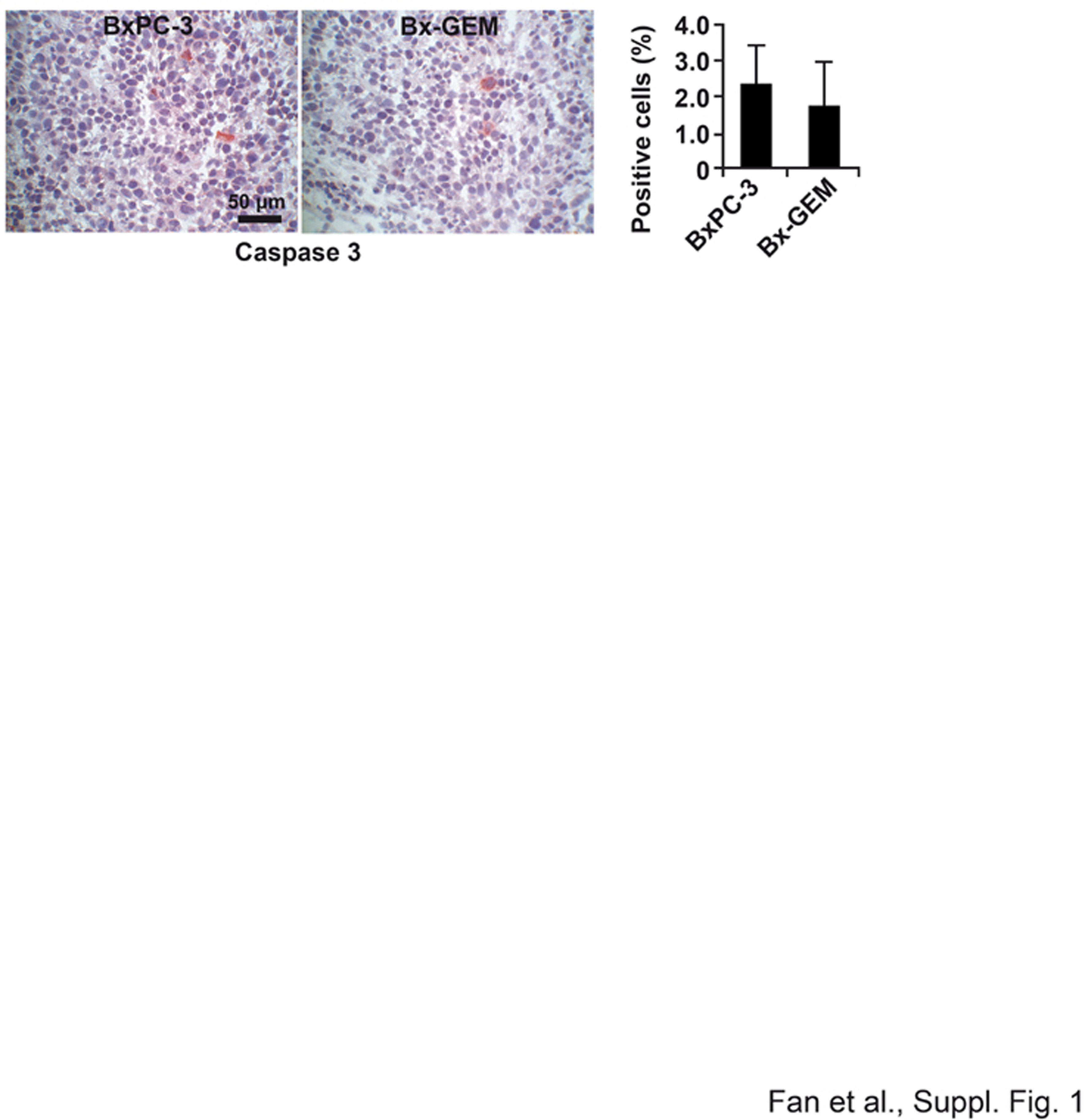

Supplement: Supplementary Figure 1 [file cddis2016157x2.tif]
