# Supplementary material for: Continuous exposure of pancreatic cancer cells to dietary bioactive agents does not induce drug resistance unlike chemotherapy
Source: Cell Death Dis. 2016 Jun 2;7(6):e2246–. doi: 10.1038/cddis.2016.157 (PMC5143386; doi:10.1038/cddis.2016.157)
Supplement: Supplementary Figure 1 Legend [file cddis2016157x3.doc]

**Supplemental Figure Legends**

**Supplemental Figure 1. Expression of the cleaved fragment of activated caspase-3 in BxPC-3 -and Bx-GEM-derived mouse xenografts.**

Tumor tissue sections from xenografts were evaluated by immunofluorescencestaining for the expression of the cleaved fragment of activated caspase-3 (brown), which is an apoptosis marker. Representative pictures at 100× magnification are shown on the left. The percentage of positive cells was counted in ten vision fields and the means ±SD are shown in the diagram on the right.
